# Notes and News

**Published:** 1889-11-23

**Authors:** 


### NOTES AND NEWS.

COLLECTED AND COMMUNICATED.

A MOST successful meeting has been held at Loughborough in aid of a convalescent home for Leicestershire. The existing home is quite inadequate, and a site having been offered in Charnwood Forest, it seems a pity not to take advantage of it and build a suitable institution. The mayor presided over the meeting, and before it broke up over £1,000 had been subscribed.

Two remarkable cases of somnambulism are reported from Berlin. A boy and girl, aged about eleven, suddenly developed somnolency. Whilst playing and in school they suddenly fall asleep; also whilst walking, standing, or speaking, so that they do not finish their sentences. If they are put to bed and afterwards awake, they try to continue the conversation which was broken off by sleep, and answer questions which were then asked them.

GREAT will be the glee of Dr. Laurie at the conversion of Dr. Lauder Brunton to his views with regard to chloroform. Dr. Brunton was only a short time in Hyderabad before he telegraphed home that he had seen 270 experiments, which all confirmed the conclusions of the Hyderabad Chloroform Commission, on whose correctness the *Lancet* threw doubt. The Nizam of Hyderabad will now feel that his patronage of medical science has not been in vain, and Dr. Laurie will be repaid for his labours by the recognition of their value.

THE annual report of the Royal Isle of Wight Infirmary is issued for the year. The total income from all sources amounted to £2,551 8s. 1d., and the total expenditure to £2,572 7s. 1d., so that there remained a small balance of £20 19s. due to the treasurer. The member for the island is in future to be a vice-chairman of this institution. The report is in every way satisfactory; the building has been enlarged, the nursing has been improved, and a special mark of Royal favour has been conferred on the infirmary this year.

SYDNEY is going to spend £34,000 on sewerage works, and it is hoped the example will be followed by Melbourne; in both towns the death-rate from typhoid is enormous. In every known case in which such system of sewerage has been adopted, the mortality from the above-mentioned disease has decreased. In Adelaide it fell from 27·6 to 9·18; in Frankfort-on-Maine, from 40 per 100,000 of the population to 5; in Munich, from 242 to 83; in Hamburg, from 48 to 10 per 1,000 of the total deaths; and in Dantzic, from 26·6 to 2·3. The population of Sydney and suburbs now amounts to nearly 400,000, but the sewers are designed for a prospective population of some 750,000 persons. The engineer who has devised these extensive works is Mr. George H. Stayton.

THE expulsion of the religious orders from the hospitals of France is being followed as an example by Italy. The "Concettini," or Friars Hospitalers, have been turned out of the Santo Spirito Hospital at Rome. The Superior-General of the order has published a protest, in which he says that during the last thirty-two years fifty of the religious have lost their lives through their duty to the sick, and surely there ought to be some recompense?

SIR SYDNEY WATERLOW, the ever-generous treasurer of Barts., is again to the fore with a munificent present to the people. At a meeting of the London County Council a letter was read from Sir Sydney Waterlow offering as a free gift a picturesque tract of land belonging to him at Highgate as a public park, together with a donation of £6,000 in cash. Lord Rosebery mentioned that on the spot the house of Nell Gwynne formerly stood. The offer was accepted amid applause, and London will shortly be the richer by one more park, and undoubtedly the healthier also.

THE seventeenth annual report of Mrs. Black's Cottage Hospital is very cheering, the number of attendances during the past year for dressing sores having reached the number of 6,433; 423 new patients have been admitted during the year, and there remained on the books at the close of the previous year 93, making a total of 516 who have sought the benefits of the hospital. The hospital is intended for out-patients, but during the year just closed five destitute patients have been received and boarded out at 12s. each per week, at the expense of the hospital. This institution is at Southampton, and is unique of its kind, being specially for cases of ulcerated legs and eczema.

HERE is Dewsbury proving grateful to its doctors! Such conduct must be chronicled. The Board of Management of this institution have held a monthly meeting, and some conversation took place upon a very dangerous and important operation successfully performed upon a patient from Liversedge, an ovarian tumour, 40lb. in weight, having been removed, and the patient now approaching convalescence. It was unanimously resolved, on the motion of the President, seconded by Mr. W. Sharrock, and supported by Messrs. Ingram and Pickersgill: "That, in the opinion of this board, the time has come when the very valuable and unselfish labours of the honorary medical staff in this institution should have special recognition; that a sub-committee be appointed to consider how this may best be done, and that a report on the subject be submitted to the next monthly meeting."

DR. OGLE, senior physician to the Derby Infirmary, has received the following letter from Miss Nightingale: "Oct. 30, '89. Dear Sir. —— has sent me 'a copy of the proposed alterations in the rules of your Derby Infirmary, and has told me that it is your wish that I should look over them and make any suggestion,' and 'return them to you before the annual meeting on the 31st.' I have taken your wish as a command, and now return the 'copy' with pencil suggestions, which I humbly offer, regretting that they are not more worthy, and that I, under the heavy pressure of work and illness, had not more time allowed me. I also enclose a sheet of remarks to explain the suggestions. I must cry you mercy that I did not send them by yesterday's post, but heavy business all day rendered it impossible. I hope that they may be of some little use to you. You have my very best wishes for the highest success of your valuable institution.—Pray believe me, ever your faithful servant, FLORENCE NIGHTINGALE." Miss Nightingale must have been amused to find that the new rules did not insist on a trained matron, a point on which she has so often given an emphatic opinion.

The following vacancies are announced this week, the amount of the salary in each case being given after the name of the institution:

**Resident Medical Officers.**

Pendlebury Hospital for Children. Junior Medical Officer—£80, board, &c.
North-Eastern Hospital for Children. Junior House Surgeon—£60, board, &c.
Warneford Hospital. House Surgeon—£100, board, &c.
Wolverhampton Hospital. Resident Assistant—Board, &c.
Halifax Infirmary. Senior House Surgeon.—£80, board, &c.
Halifax Infirmary. Assistant House Surgeon. — £50, board, &c.

**Non-Resident Medical Officers.**

Woodstock Union. Medical Officer—£75.
London Hospital, Whitechapel. Surgical Registrar—£100.
King's College, London. Sambrooke Surgical Registrar.
Great Northern Central Hospital. Ophthalmic Surgeon.

A sale of work in aid of Coldstream Cottage Hospital was lately held, under the superintendence of the Countess of Horne. Several ladies of the neighbourhood, assisted by the nurses, presided at the stalls, and the result was the taking of £88, which goes to endow a bed at the hospital.

The extension scheme of Cardiff Infirmary goes on apace, and some munificent donations are promised. Lord Tredegar gives £300, Mr. C. Thompson £500, Lord Windsor £200, Mr. J. Cory £250, Mr. J. Gaskell £500, Colonel Hill £200, and many other donors are to the fore. Cardiff should be proud of its hospital committee and friends.

The annual meeting of Salop Infirmary took place on the 14th, followed by the usual function which seems so dear to the dwellers in Shrewsbury. A want of funds and of various improvements in furniture, etc., was recorded, and the nurses were congratulated on their improvement. Mr. Harold B. Macleod was appointed house surgeon in place of Mr. Harris, resigned.

The matron of the Torbay Hospital has been interviewed, and made to deliver a perfect oration in favour of the hospital. She complains that the hospital is much too old for present-day requirements, and pleads for further support from the people of the town, so that necessary improvements and enlargements may be made. Miss Scholfield explained that if every inhabitant of Torquay would give a penny a month an income of £1,500 would be secured to the hospital, an income which would supply all the needs.

The new Lunacy Amendment Act needs careful consideration by all doctors. Besides the two medical certificates which were formerly sufficient, it is now necessary that any person, other than a pauper, alleged to be insane, must be examined by a County Court judge or justice of the peace specially appointed, or by some other competent tribunal, before he or she can be received into any private asylum. With regard to the case of paupers, in future all orders for a patient's reception must be signed by a justice of the peace, and the power given by the old Act, 16 and 17 Vic., cap. 97, to officiating clergymen and relieving officers to sign orders for the reception of pauper patients into an asylum is repealed.

The question of "professional pressure," we have reason to know, is exercising the minds of many general practitioners very seriously. An important communication from a correspondent, who is himself in family practice, is unavoidably held over until next week. Whatever may be the case with other learned professions, there is no doubt whatever that the medical man's life is one of incessant and anxious toil, and of small pecuniary rewards. The public may think it an advantage to have plenty of cheap doctors. But it is as true of cheap doctoring as of other cheap things that quality is generally in strict proportion to price.

To the energy of Lady Rosebery is greatly due the excellent first annual report just issued by the Queen Victoria Jubilee Institute for Nurses at Edinburgh. The committee engaged the double flat, 5, North Charlotte-street, as a temporary training home, and in December engaged Miss Peter as superintendent. She began her work in Edinburgh on April 1, with three nurses, and five others have been successively added. In addition to their hospital, maternity, and district training, the nurses have attended a course of lectures on hygiene, and lectures on food and practical cooking lessons. One nurse has already been engaged by the Kilmarnock Nursing Association for the Sick Poor. Another begins work at Wemyss in November. A third has been placed by arrangement with Miss Cowan in Fountainbridge. The medical officers of the Royal Dispensary, Richmond-street, have arranged for part services of a nurse. The total receipts have amounted to £2,360 15s. 9d., and the total expenditure to £671 14s., leaving a surplus of £1,689 1s. 9d., which may be regarded as the nucleus of a building fund. Lady Rosebery was present at the annual meeting, and has founded a Needlework Guild to make garments for the poorer patients.

On the 30th of this month a meeting of the medical profession in Birmingham will be held, to consider a report of a sub-committee on the subject of the abuse of medical charity. It is intended to specially point out: (*a*) That the original purpose of our medical charities has been widely departed from. (*b*) That as a result of this departure the demands made upon them are greater than they can adequately meet, and are increasing from year to year. (*c*) That a constantly increasing proportion of the community depends upon charitable medical relief in times of sickness, rather than on providence and self-help, and that this is unjust to the subscribing public, injurious to the recipients themselves, and an imposition upon the medical profession. (*d*) That in order to bring about a better state of things the voluntary hospital system needs to be reorganised, so that, on the one hand, it may work harmoniously with and supplement the Poor Law system, and, on the other hand, it may encourage, and not hinder, as it now does, the development of provident institutions for the medical treatment of the industrial classes. The report dwells upon the injustice which is done to the medical profession, and makes the following suggestions in the direction of reform: A large part of the work which is now done in the out-patient department of the hospitals should be done by provident dispensaries. Those who are too poor to belong to provident dispensaries should be treated, under all ordinary circumstances, through the agency of the Poor Law. The voluntary hospitals, co-operating with the provident and the Poor Law systems, should treat cases of severity, difficulty, or importance, such as would be properly referred to them for the special advantages of hospital treatment. In order that medical education should not suffer through such restriction of the work of the voluntary hospitals, the Poor Law infirmaries and the provident dispensaries should also be utilised for this purpose. It is not desirable that radical changes of any kind should be suddenly introduced. Careful and impartial enquiry as to the existing state of things must precede any practical attempt to establish a better, and no attempt can succeed unless there be a general agreement on the part of the several institutions and public bodies which are concerned. A conference between representatives of the voluntary hospitals, of the board of guardians, of the Hospital Saturday Committee, of the existing provident dispensaries, of the Charity Organisation Society, and of the general body of the medical profession, is the first thing to be desired.

